# ANCHOR: A Technical Approach to Monitor Single-Copy Locus Localization *in Planta*

**DOI:** 10.3389/fpls.2021.677849

**Published:** 2021-07-06

**Authors:** Anis Meschichi, Mathieu Ingouff, Claire Picart, Marie Mirouze, Sophie Desset, Franck Gallardo, Kerstin Bystricky, Nathalie Picault, Stefanie Rosa, Frédéric Pontvianne

**Affiliations:** ^1^Department of Plant Biology, Swedish University of Agricultural Sciences, Uppsala, Sweden; ^2^Université de Montpellier, DIADE, Montpellier, France; ^3^CNRS, Laboratoire Génome et Développement des Plantes (LGDP), Université de Perpignan Via Domitia, Perpignan, France; ^4^Institut de Recherche pour le Développement, DIADE, Montpellier, France; ^5^iGReD, CNRS UMR 6293, Université Clermont Auvergne, INSERM U1103, Clermont–Ferrand, France; ^6^NeoVirTech SAS, 1 Place Pierre Potier, Toulouse, France; ^7^Laboratoire de Biologie Moléculaire Eucaryote (LBME), Centre de Biologie Intégrative (CBI), CNRS, UPS, University of Toulouse, Toulouse, France

**Keywords:** chromatin, nuclear organization, real-time imaging, microscopy, single-locus analysis, chromatin mobility

## Abstract

Together with local chromatin structure, gene accessibility, and the presence of transcription factors, gene positioning is implicated in gene expression regulation. Although the basic mechanisms are expected to be conserved in eukaryotes, less is known about the role of gene positioning in plant cells, mainly due to the lack of a highly resolutive approach. In this study, we adapted the use of the ANCHOR system to perform real-time single locus detection *in planta*. ANCHOR is a DNA-labeling tool derived from the chromosome partitioning system found in many bacterial species. We demonstrated its suitability to monitor a single locus *in planta* and used this approach to track chromatin mobility during cell differentiation in *Arabidopsis thaliana* root epidermal cells. Finally, we discussed the potential of this approach to investigate the role of gene positioning during transcription and DNA repair in plants.

## Introduction

In eukaryotes, genetic information is encoded in the chromatin, a complex structure composed of DNA packed around an octamer of histones in the nucleus. Chromosome territories form large compartments in the nucleus, themselves containing chromatin domains harboring different epigenetic signatures (Nguyen and Bosco, 2015; Pontvianne and Grob, 2020; Santos et al., 2020). In these domains, the positioning and accessibility of genes are very dynamic in response to several key biological processes that include gene transcription, genome replication, and DNA repair. Fluorescence *in situ* hybridization (FISH) approaches, such as padlock-FISH, enable to detect a single-copy locus using the fixed plant material (Feng et al., 2014). However, imaging techniques using non-living organisms are insufficient to track spatial and temporal dynamics of loci. The live-cell imaging approaches allow gene positioning visualization during these different processes, providing key elements for their understanding (Dumur et al., 2019; Shaban and Seeber, 2020).

Microscopic detection of genomic loci in plants is possible through the use of different strategies, such as zinc-finger-based imaging, transcription activator-like effectors (TALEs), and CRISPR/Cas9 (Lindhout et al., 2007; Fujimoto et al., 2016; Khosravi et al., 2020). Unfortunately, these techniques have been restricted to follow the dynamics of highly repeated regions (i.e., centromeric repeats, telomeric sequences, and ribosomal RNA genes). Monitoring a single locus in living plants is possible due to the addition of *lacO* motifs to which the transcription factor LacI, fused to a fluorescent protein, can bind (Kato and Lam, 2003; Fang and Spector, 2007). Live-cell imaging of *Flowering Locus C* (*FLC*) alleles associated with *lacO* (*FLC-LacO*) could be performed to demonstrate that *FLC-LacO* repression during vernalization provokes their physical clustering (Rosa et al., 2013). In addition, the Tet repressor protein fused to a fluorescent protein could also be used to label a genomic region containing numerous Tet operator sequences (Matzke et al., 2005). In both cases, amplification of the signal is directly linked to the multiplicity of the targeted sequences. However, these repetitions often affect local chromatin organization and can trigger silencing of the reporter gene (Watanabe et al., 2005). Thus, a standardized and robust technique for tracking the dynamics of a single locus is still not available.

**Figure 1:**
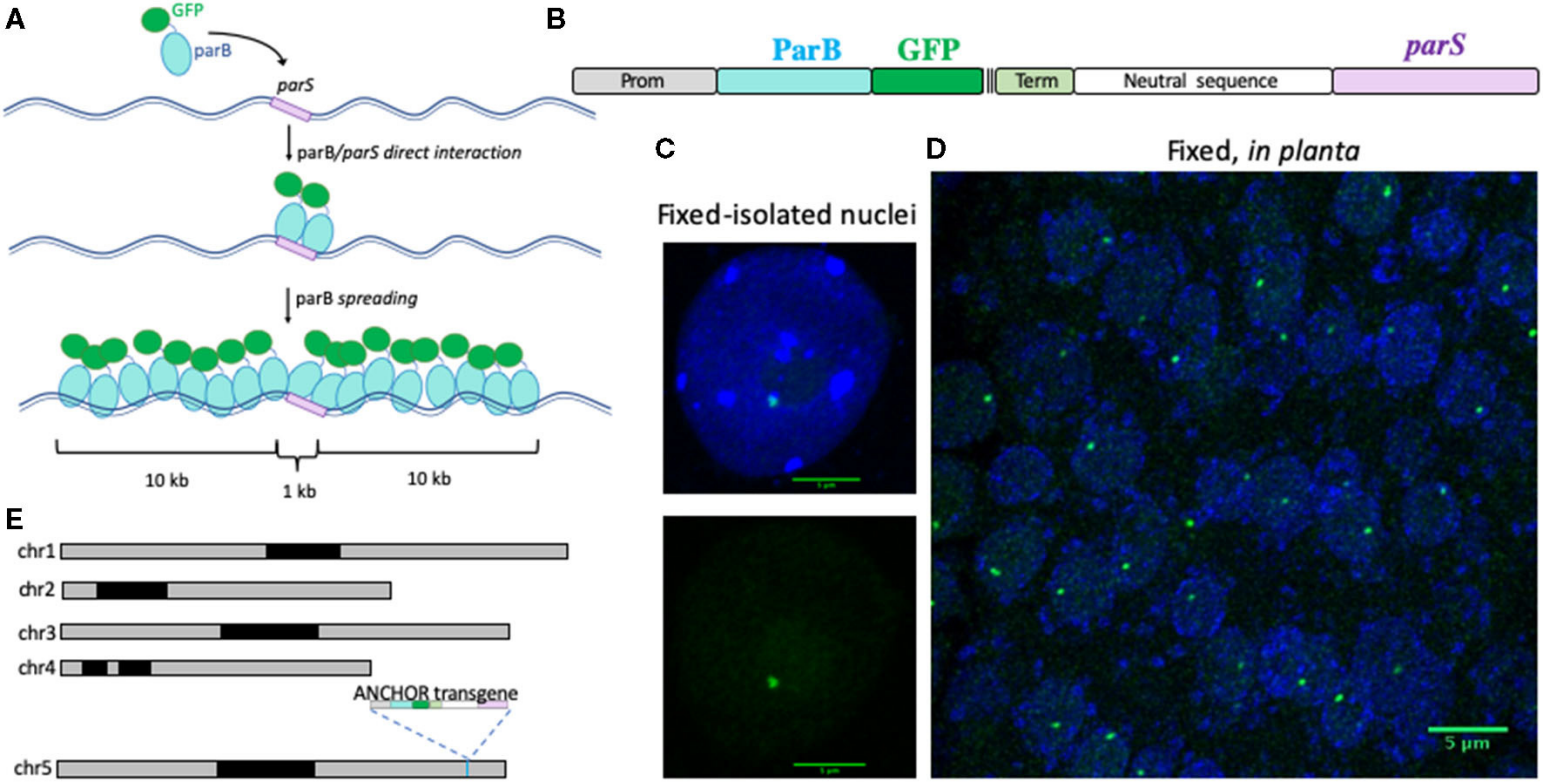
Description of the ANCHOR system *in planta*. **(A)** Schematic representation of the ANCHOR system. ParB proteins fused to GFP can directly bind to *parS* sequence as a dimer. *parS*-ParB interactions provoke a conformational change in ParB proteins that induce their oligomerization along the flanking genomic region. **(B)** Cassette used to transform *Arabidopsis thaliana* Col-0 plants to test the ANCHOR system *in planta*. A strong and ubiquitous promoter is used to express the ParB protein fused to GFP and three FLAG tags. After a terminator sequence, a 1.5 kb-long spacer sequence has been added to separate the ParB:GFP open reading frame and the 1 kb-long *parS* sequence. Detection of a *parS*-ParB:GFP focus (green) in an isolated leaf nucleus **(C)** and in fixed root tissues **(D)** of *A. thaliana* plants containing the ANCHOR cassette described in **(B)**. Nuclear DNA is labeled with DAPI (blue). Bar = 5 μm. **(E)** Position of the transgene in the ANCHOR line T2F in the *Arabidopsis* genome using nanopore sequencing. The transgene presented in **(B)** is inserted on chromosome 5, position 23.675.998 bp.

The ANCHOR system is a DNA-labeling tool derived and optimized from chromosome partitioning complex of bacteria. A single-copy of *parS*−1-kb-long fragment—serves as a binding platform for ParB proteins (Dubarry et al., 2006). Natural *parS* sequence is composed of four canonical inverted repeat sequences that are bound *via* the helix-turn-helix motif present in ParB (Funnell, 2016). Upon binding, oligomerization of ParB proteins then propagates over the *parS* sequence and adjacent DNA (Figure 1A). Importantly, oligomerized ParB proteins are loosely associated and can be displaced transiently and easily upon transcription or DNA repair (Saad et al., 2014). This phenomenon is also described as the caging step (Funnell, 2016). This system has been adapted successfully to monitor a unique locus in living yeast and human cells using a fluorescent-tagged ParB (Germier et al., 2017). This approach is also able to visualize DNA viruses in human cells (Komatsu et al., 2018; Mariamé et al., 2018; Blanco-Rodriguez et al., 2020; Gallardo et al., 2020; Hinsberger et al., 2020). In this study, we demonstrated that the ANCHOR system can also be used to visualize a single locus in fixed and living plant tissues. Using this approach, we also revealed that chromatin mobility is distinct in differentiated cells compared with meristematic cells of plants.

## Materials and Methods

### Plant Materials and Growth Conditions

**Figure 2:**
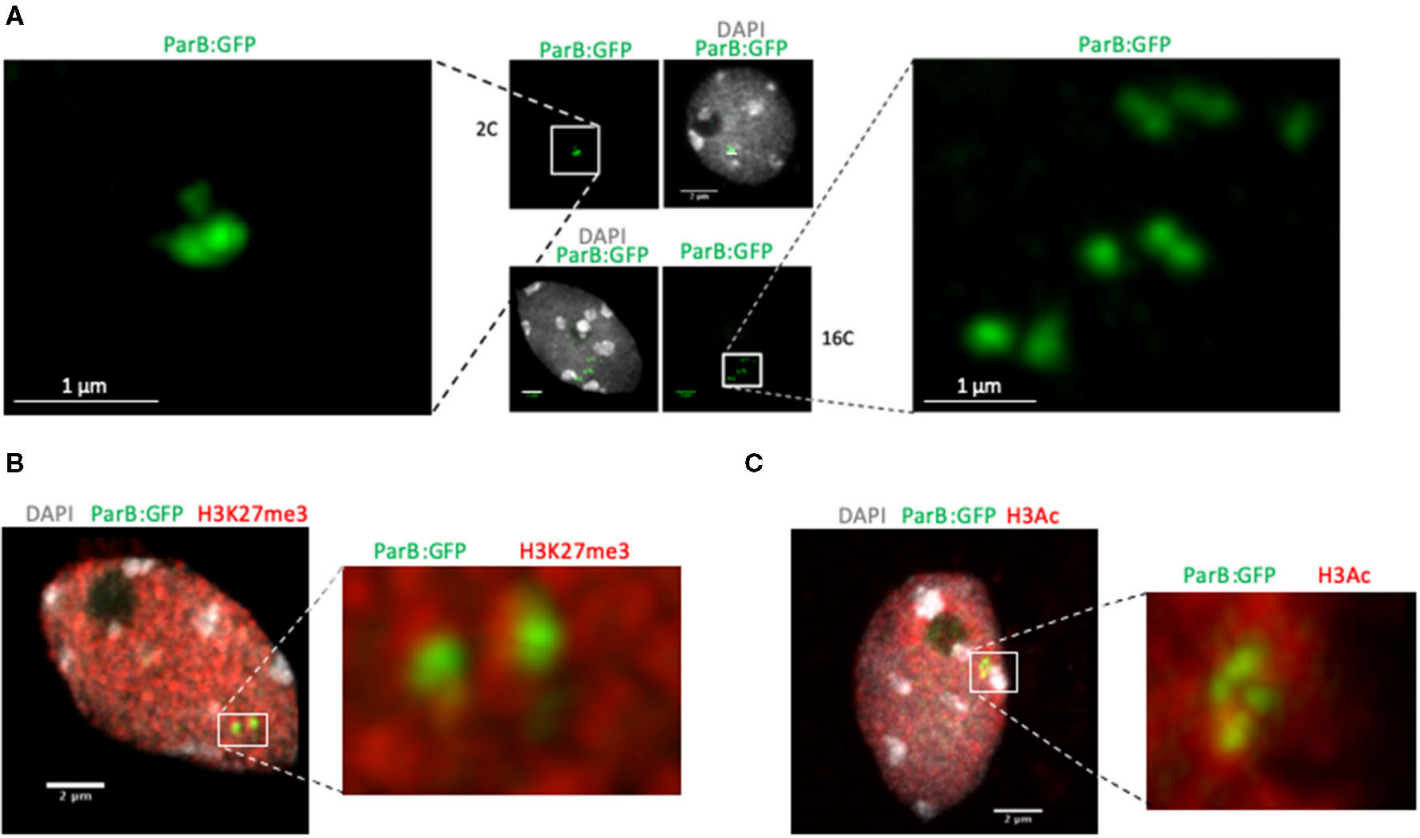
Detection of *parS*-ParB foci in cells with different ploidy levels and after immunolocalization experiments. **(A)** Detection of *parS*-ParB:GFP foci (green) in fixed and sorted nuclei according to their ploidy levels by fluorescent-assisted cell sorting. Nuclear DNA is labeled with DAPI (gray). Enlarged view of the *parS*-ParB:GFP foci is presented to facilitate signal visualization. Bar = 1 μm. **(B,C)** Detection of *parS*-ParB:GFP foci (green) and posttranslationally modified histones (red) in fixed and isolated nuclei *from A. thaliana* Col-0 plants T2F. The image corresponds to a confocal 2D stack. Nuclear DNA is labeled with DAPI (gray). Trimethylated H3K27 signals are shown in **(B)**, while acetylated H3 are shown in **(C)**. Enlarged views of the *parS*-ParB:GFP foci are presented to facilitate signal visualization. Bar = 2 μm.

*Arabidopsis thaliana* ecotype Col-0 was used in this study. *lacO*/LacI line used was obtained from the study by Matzke et al. (2005). To test the ANCHOR system, *A. thaliana* (Col-0) plants were transformed by agroinfiltration using the floral dip protocol (Clough and Bent, 1998), using *Agrobacterium tumefaciens* GV3101 strain. Transformants were grown on soil and sprayed with Basta herbicide for selection (10 mg/L). All the plant materials used here was grown in control growth chambers on soil at 21°C with a daylight period of 16 h/day. The transformant 2F (T2F) line was crossed to Col-0 wild-type plants expressing the histone variant H2A.W fused to a red fluorescent protein (RFP) (Yelagandula et al., 2014). The T2F line used in this study is heterozygote for the ANCHOR transgene, except in the data shown in Figure 2, where homozygous lines have been used.

For *in vitro* growth, seeds were surface sterilized in 5% v/v sodium hypochlorite for 5 min and rinsed three times in sterile distilled water. Seeds were stratified at 4°C for 48 h in the darkness and plated on the Murashige and Skoog (MS) medium. Seedlings were placed in a growth cabinet (16 h light, 22°C) for 1 week in a vertically oriented Petri dish before imaging.

### Plasmid Construction

A cassette allowing the expression of ParB has been synthetized by GenScript (USA). The nature and sequences of the ANCHOR system and the property of NeoVirTech SAS are confidential. The cassette was cloned into the pEarleyGate302 vector (Earley et al., 2006).

### Nanopore Sequencing

Genomic DNA preparation was performed as previously described by Picart-Picolo et al. (2020). Library preparation was performed using the 1D genomic DNA with ligation kit SQK-LSK109 (Oxford Nanopore Technologies, UK), following the instructions of the manufacturer. The R9.5 ONT flow-cell FLO-MIN106D (Oxford Nanopore Technologies, UK) was used. We obtained 1.93 GB of sequences (11 × coverage) with an average read length of 3,675 kb for ANCHOR T2F line. ONT reads mapping the transgene were mapped, filtered, and aligned using Geneious® software (Kearse et al., 2012).

### Cytogenetic Analyses

For cytogenetic analyses, nuclei were isolated from 3- or 4-week-old plants as previously described (Pontvianne et al., 2012). In brief, fresh leaves were fixed in 4% formaldehyde in Tris buffer (10 mM Tris–HCl at pH 7.5, 10 mM EDTA, and 100 mM NaCl) for 20 min and then chopped with a razor blade in 0.5 mL of LB01 buffer (15 mM Tris–HCl at pH 7.5, 2 mM NaEDTA, 0.5 mM spermine, 80 mM KCl, 20 mM NaCl, and 0.1% Triton X-100). The lysate was filtered through a 30-μm cell strainer (BD Falcon, USA), and 12 μL of sorting buffer (100 mM Tris–HCl at pH 7.5, 50 mM KCl, 2 mM MgCl_2_, 0.05% Tween-20, and 5% sucrose) was added per 3 μL of cell/nuclei suspension (Pontvianne et al., 2012) and spread on a polylysine slide. After air-drying, the samples were postfixed in 2% formaldehyde in phosphate buffer (PBS) for 5 min and then washed twice with water before being air-dried. The slides were then mounted in Vectashield at 1 μg/mL of DAPI and sealed them with nail polish.

**Figure 3:**
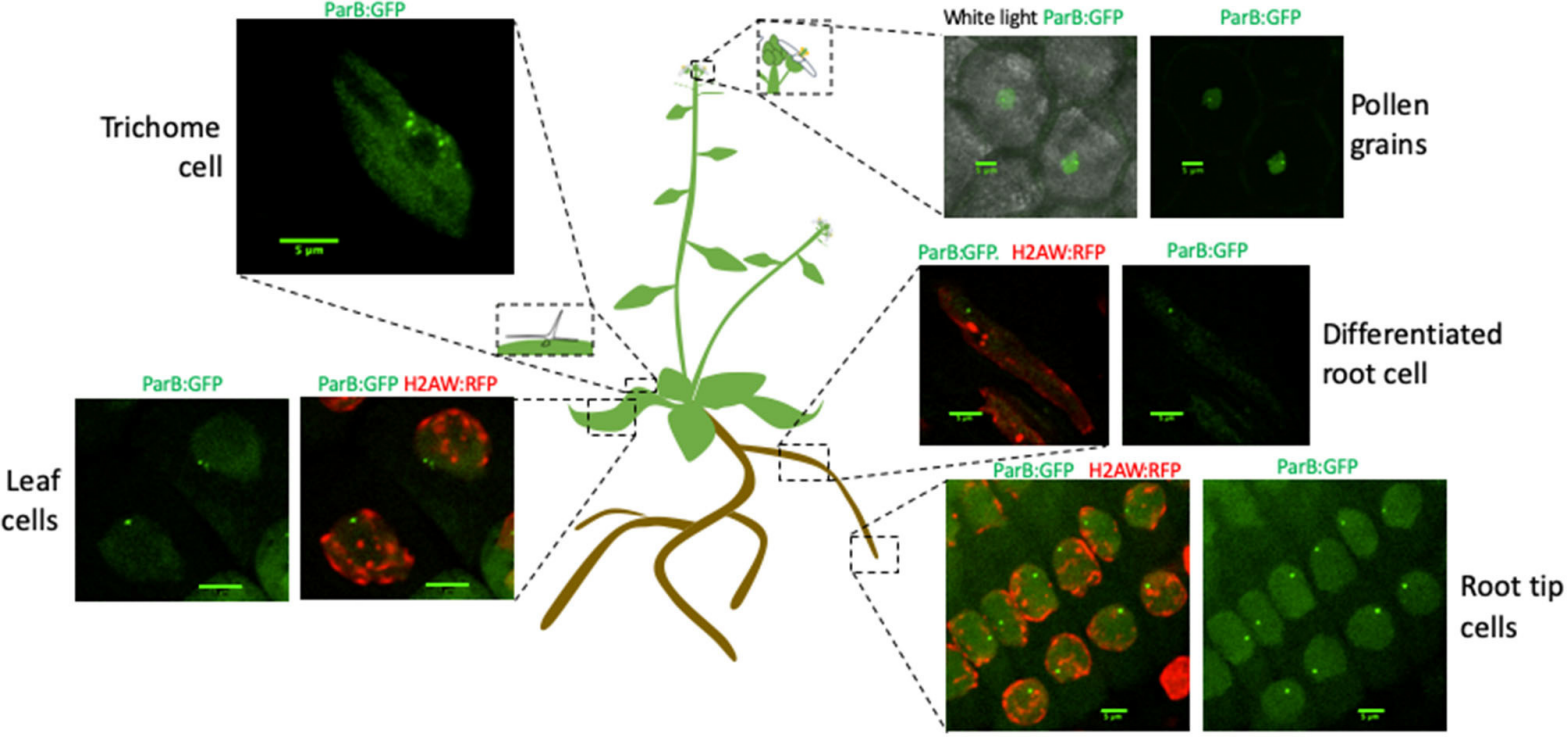
ANCHOR system is suitable to monitor a single-copy locus in live cell and different tissues. Schematic representation of an *A. thaliana* plant illustrating the different tissues in which *parS*-ParB:GFP signals have been detected by live-cell imaging. ParB:GFP signals are shown in green and H2A.W:RFP is shown in red. Scale bars = 5 μm.

**Figure 4:**
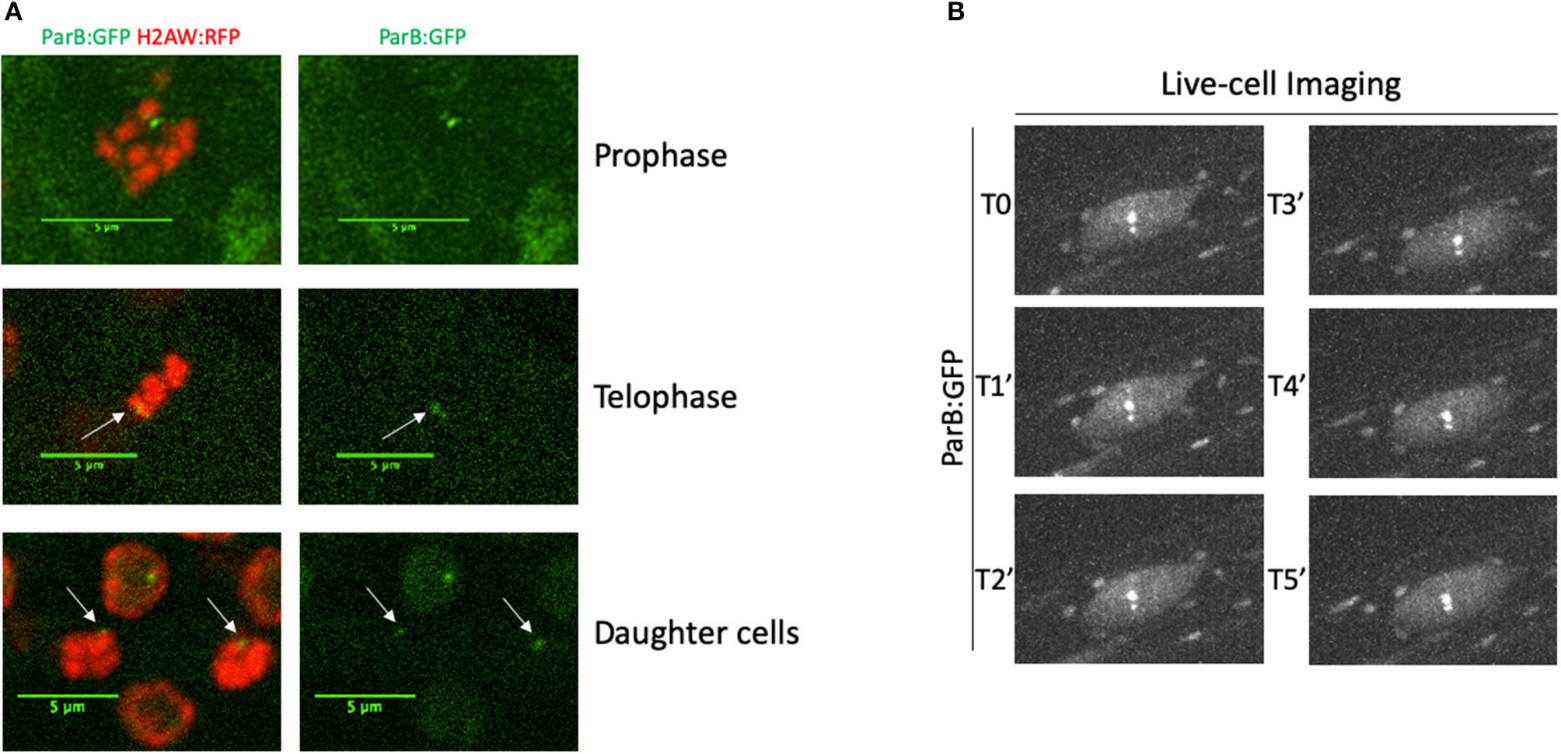
Monitoring parS-ParB:GFP in live cell during mitosis or during a time-course. **(A)** Detection of *parS*-ParB:GFP foci (green) and H2A.W:RFP (red) in mitotic cells. Scale bars = 5 μm. **(B)** ANCHOR system enables time-lapse tracking of a single locus in live roots by confocal imaging. Time-lapse acquisition of *parS*-ParB:GFP signals (gray) in an endoreplicated root cell over 5 min.

Nuclei with different levels of ploidy were isolated as described by Pontvianne et al. (2016), except that propidium iodide was used to stain the nuclei, together with RNase to a final concentration of 10 μg/mL. A S3 cell sorter (Biorad, USA) with 488 nm and 561 nm 100 mW dual-lasers was used to sort the nuclei. Immunolocalization experiments were performed as described previously (Durut et al., 2014) using anti-H3K27me3 or anti-H3Ac antibodies (Abcam, USA) to a 1/1,000 dilution. Zeiss LSM 700 confocal was used to generate images as shown in Figure 1, while Zeiss LSM 800 with an Airyscan module was used to generate images as shown in Figures 2–4A with a 63 × objective, N.A. 1.4 and pixel size of 0.028 × 0.028 × 0.160 μm^3^. Live-cell imaging shown in Figure 4B were performed using a spinning disk Zeiss Cell Observer equipped with a high-speed Yokogawa CSUX1 spinning disk confocal, an ORCA-flash 4.0 digital camera Hamammatsu (Japan) and a 40 × water objective N.A. 1.2. Green fluorescent protein (GFP) was excited at 488 nm.

### Live-Cell Imaging

**Figure 5:**
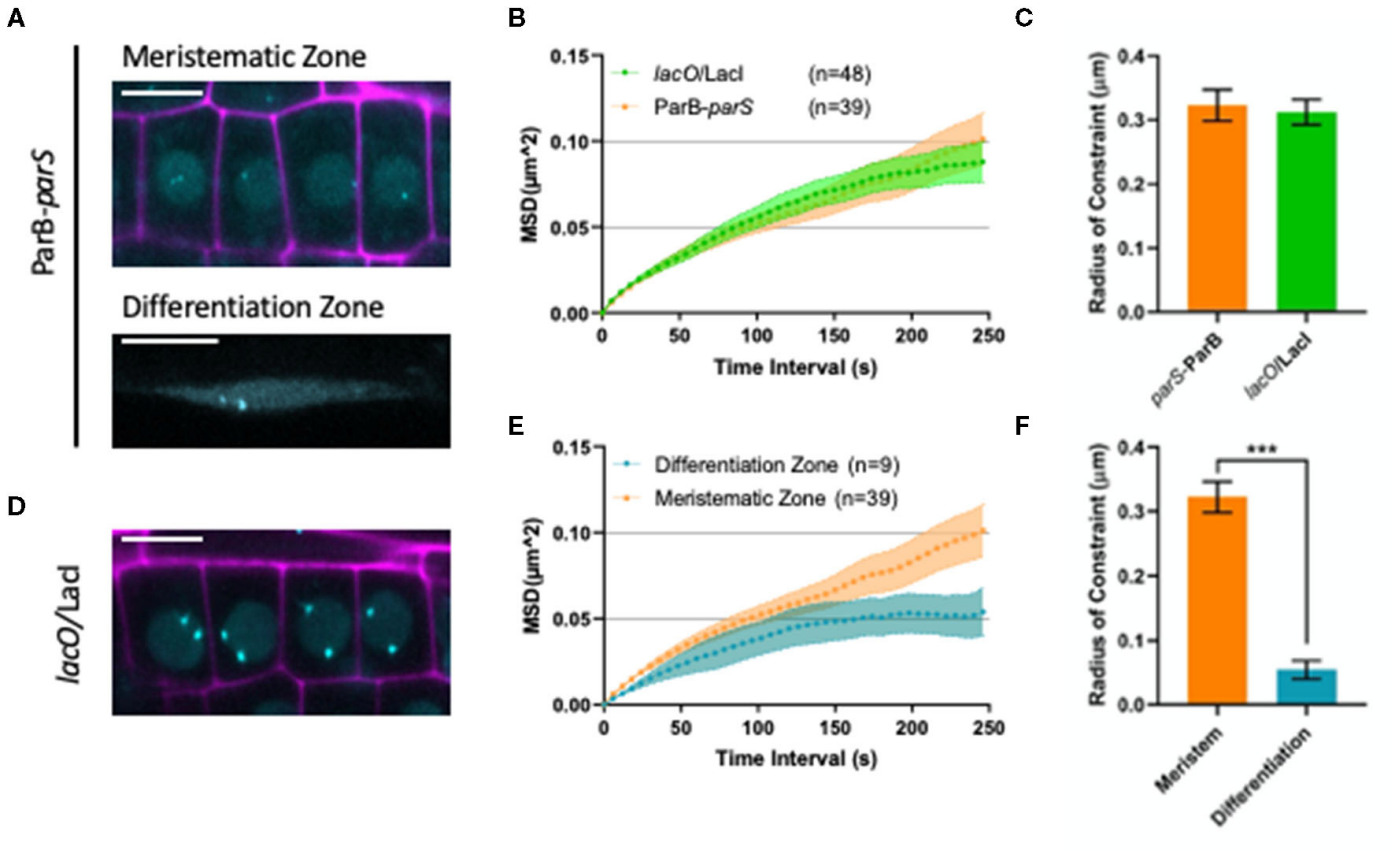
Analyzing chromatin mobility using the ANCHOR system. **(A)** Representative images of ParB-*parS* line in meristematic (upper panel) and differentiation zone (bottom panel)showing nuclear signal with spots (cyan). Propidium iodide (PI) staining is shown in magenta. Bars = 10 μm. **(B)** The MSD analysis for *lacO*/LacI and ParB-*parS* lines based on time lapse experiment of nuclei in the meristematic zone. The values represent means ± SEM from 48 and 39 cells, respectively. **(C)** Calculated radius of constraint for MSD curves depicted in **(B)**. The values represent means ± SEM. Student's *t-*test, ***P < 0.001. **(D)** Representative image of *lacO*/LacI line in meristematic region showing nuclear signal with spots (cyan). PI staining is shown in magenta. Scale bar = 10 μm. **(E)** The MSD analysis for ParB-*parS* lines based on time-lapse experiments of nuclei in the meristematic and differentiated zone. 3D stacks were taken at 6 s intervals for 5 min. The values represent mean ± SEM from 39 and 9 cells, respectively. **(F)** Calculated radius of constraint for MSD curves depicted in **(E)**. The values represent means ± SEM.

In Figure 5, time-lapse imaging of *A. thaliana* roots has been carried out using a Zeiss LSM 780 confocal microscope with a 63 × water immersion objective (1.20 N.A.). For visualization of root cell contours stained with propidium iodide, an excitation line of 488 nm was used, and the signal was detected at wavelengths of 580–700 nm. For the observation of GFP expression, we used a 488-nm excitation line and a band-pass filter of 505–550 nm. For all experiments, the images were acquired every 6 s, taking a series of three optical sections with a Z-step of 2 μm for 5 min. Each movie has a format of 512 × 512 pixels and a 3 × zoom factor.

The 7-day-old seedlings were mounted in water, or propidium iodide, between slide and cover slip and sealed with a 0.12-mm-thick SecureSeal adhesive tape (Biorad, USA), to avoid root movements and drying during imaging.

### Mean Square Displacement Analysis

All the movies have been analyzed with Fiji software (NIH, Bethesda, MD, USA, http://rsb.info.nih.gov/ij/) and with the plugin SpotTracker 2D (obtained from www.epfl.ch/sage/soft/spottracker). The mean square displacement (MSD) analysis was performed as described by Meschichi and Rosa (2021). All quantitative measurements represent averages from at least nine cells. From the MSD plot, we calculated the radius of constraint by the square root of the plateau of the MSD curve multiplied by 5/4. Data sets were tested for normality using the Shapiro–Wilk test. The parametric analyses were done using the standard Student's *t-*test to determine the statistical significance of the results. For the statistical analysis, we used the GraphPad Prism 8.3 software.

## Results

### Development of the ANCHOR System

Our aim was to adapt and facilitate the use of the ANCHOR system in plants. We, therefore, combined the two elements of the ANCHOR system (ParB and its target sequence *parS*) into a single transgene. A *ParB* gene, whose coding sequence has been optimized for *A. thaliana*, was fused in a frame to a GFP and triple FLAG-tag (ParB:GFP:3XFLAG) to allow detection in living and fixed nuclei (Figure 1B). *ParB:GFP:3XFLAG* expression was placed under the control of a promoter allowing ubiquitous expression. At the 3′ end of the ParB construct, we added the 1-kb-long ParB target sequence *parS* separated by a 1.5-kb-long spacer sequence to prevent the potential interference of *ParB* gene transcriptional activity. Such design allows a rapid selection of transgenic plants containing the two linked ANCHOR elements. In addition, detection of *parS*-ParB:GFP signals would suggest that *ParB:GFP* transcription is possible even in the case of local caging of ParB:GFP proteins.

Wild-type Col-0 plants were transformed with the transgene and selected using Basta herbicide by spraying. Fixed nuclei isolated from eight different T1 transformants revealed the presence of *parS*-ParB:GFP foci in five of them (Figure 1C). To test the robustness of the detection approach, we then analyzed the entire root-tip from one ANCHOR line comprising a single-copy insertion at generation T2 (T2F; Figure 1D). One *parS*-ParB:GFP signal was detectable in almost all nuclei analyzed. Importantly, the signal-to-noise ratio is high, which allows easy detection of the specific signal (Figure 1D).

To further characterize the ability of the ANCHOR system to follow a single-locus *in planta*, it is important to know the exact location of the transgene. We performed long-read nanopore sequencing on an ANCHOR line with one single insertion (T2F) and extracted all long reads corresponding to the transgene to map its location in the genome. The sequence analyses revealed that the transgene could be located on the lower arm of chromosome 5, at position 23.675.998 bp, in an intergenic region (Figure 1E). This position is flanked by a region enriched in active chromatin marks and a region enriched with histone 3 trimethylated lysine 27 (H3K27me3), a repressive mark deposit by the polycomb repressive complex 2 (PRC2) (Supplementary Figure 1) (Sequeira-Mendes et al., 2014).

### Detection of *parS*-ParB *Foci* in Fixed Cells

As shown in Figure 1D, one unique focus was usually detected in root tip cells, sometimes appearing as a doublet. Since the ANCHOR system is based on protein aggregation, we checked whether analyzing ANCHOR signals in endoreplicated cells would lead to an increased number of detected foci. We isolated 2C, 4C, and 16C cells by fluorescent-assisted cell sorting after propidium iodide labeling and RNase treatment. We stained sorted nuclei with DAPI and observed *parS*-ParB:GFP signals in sorted nuclei. We could see a higher amount of *parS*-ParB:GFP signals in sorted nuclei presenting a higher endoreplication rate (Figure 2A and Supplementary Figure 2A). Although these data suggest that the ANCHOR system is suitable to detect multiple loci simultaneously, additional experiments are required to fully demonstrate that this reporting system does not lead to aberrant locus aggregation.

In the T2F line, the transgene is located on an arm of the chromosome 5, in a region enriched in H3K27me3 deposited by the PRC2 but flanked by a genomic region enriched with active chromatin marks (Supplementary Figure 1). Although T-DNA transgene insertion may affect this peculiar chromatin environment locally (Rajeevkumar et al., 2015), we tested the possibility to combine both immunostaining and *parS*-ParB:GFP signal detection. Immunostaining experiments were performed on isolated leaf nuclei from 3-week-old plants using either an antibody against histone 3 acetylated (H3Ac) active mark or H3K27me3 repressive mark. As expected, the tested histone marks and *parS*-ParB:GFP signals are excluded from heterochromatic foci stained by DAPI, corresponding to the centromeric, pericentromeric, and nucleolus organizer regions (Figures 2B,C). Although no clear overlap could be detected between *parS*-ParB:GFP signals and H3K27me3 marks, at least partial overlap can be seen between *parS*-ParB:GFP signals and H3Ac marks (Figures 2B,C and Supplementary Figure 4). This result is expected since active transcription is necessary to produce ParB:GFP proteins. Although we cannot conclude about the specific chromatin state surrounding the transgene insertion site in T2F, this experiment demonstrates our ability to detect *parS*-ParB:GFP signals and immunodetection approach simultaneously.

### Detection of *parS*-ParB *Foci* in Live-Cell Imaging

Previous studies demonstrate that global genome organization can be cell specific and vary during plant development (Pontvianne and Liu, 2019). Therefore, we tested our ability to detect *parS*-ParB:GFP signals in different cell-types, directly *in planta*. To allow simultaneous visualization of heterochromatin and *parS*-ParB:GFP signals directly in living cells, we crossed the T2F line with another *A. thaliana* Col-0 line expressing the histone 2A variant H2A.W, fused to the RFP (Yelagandula et al., 2014). Plants were grown on MS media directly in Petri dish compatible with confocal imaging. We analyzed several tissues, including meristematic and differentiated root cells, leaf cells, and trichome cells, and also pollen grains from plants grown on soil. We were able to detect *parS*-ParB:GFP signals in all cell types tested (Figure 3 and Supplementary Figure 3). As expected, *parS*-ParB:GFP signals are excluded from the heterochromatin area, labeled by H2A.W:RFP signals. It is noted that in certain cell types, the nuclear area can be seen due to non-associated ParB proteins that diffuse in the nucleoplasm.

The ANCHOR system does not require high DNA accessibility to allow the visualization of *parS*-ParB:GFP signals. In a highly condensed chromatin context, such as during mitosis, we could still detect *parS*-ParB:GFP signals in condensed chromosomes, even though the signal is usually less bright than in the neighboring cells (Figure 4A).

Finally, we tested our ability to perform live-cell imaging of the *parS*-ParB:GFP signals *in planta*. We analyzed *parS*-ParB:GFP dynamics in living roots using a Zeiss Cell Observer spinning disk microscope (Figure 3B). Although bleaching can alter the signal detection over time, we were able to detect the ParB:GFP signals at multiple time points and track its relative nuclear position, as reported earlier in human and yeast cells (Saad et al., 2014; Germier et al., 2017). Movies showing the detection of *parS*-ParB:GFP signals in live meristematic or elongated cells can be found in Supplementary Material (Supplementary Movies 1, 2). Altogether, our data demonstrated that the ANCHOR system is suitable for live-cell imaging *in planta*.

### Studying Chromosome Mobility Using the ANCHOR System

It is now clear that higher-order organization of the chromatin exerts an important influence on genomic function during cell differentiation (Arai et al., 2017). For instance, in *A. thaliana*, histone exchange dynamics were shown to decrease gradually as cells progressively differentiate (Rosa et al., 2013). However, how chromosomes and the chromatin fiber move during cell differentiation is not well-studied in plants. We took advantage of our ANCHOR DNA labeling system to monitor chromatin mobility changes upon cell differentiation in the T2F line. In particular, we measured the mobility of *parS*-ParB:GFP *foci* in meristematic and differentiated cells from the root epidermis (Figure 5A) through live-cell imaging using confocal microscopy and quantified the mobility using the MSD analysis (Meschichi and Rosa, 2021). Interestingly, the chromatin mobility on meristematic cells was higher than in differentiated cells (Figure 5B and Supplementary Movies 1, 2). These differences were statistically significant as shown by a much higher radius of constraint (Figure 5C). These results may support the idea that the chromatin in undifferentiated cells holds a more dynamic conformation (Meshorer et al., 2006; Rosa et al., 2013; Arai et al., 2017). However, additional experiments would be required to further validate the biological relevance of this result.

Since single-locus dynamics in plants was mostly possible through the use of the *lacO*/LacI system (Figure 5D), we thought to compare chromatin mobility in meristematic cells using the ANCHOR and the *lacO*/LacI systems. Interestingly, both methods revealed a very similar MSD curve. In fact, a MSD curve, where the maximum values asymptotically reach a plateau, indicates that chromatin moves in a subdiffusive manner, which is typical for chromosomal loci tracked in interphase nuclei (Seeber et al., 2018). Additionally, the curves resulted in comparable measurements of the radius of constraint (Figures 5E,F), showing that the chromatin environment for these two insertion lines may be similar. While the comparison of additional lines with different chromosomal locations would be interesting, the results presented here illustrate that the ANCHOR system can be used to monitor single-locus and is suitable to study chromosome organization and dynamics in plants.

## Discussion and Perspectives

In this study, we described a novel method to monitor a single-copy locus *in planta*. In comparison with existing strategies, the advantage of the ANCHOR system is the absence of repeated elements in the target sequence. This aspect is especially important in plants due to the existence of plant-specific silencing systems (Watanabe et al., 2005; Matzke et al., 2015; Grob and Grossniklaus, 2019). In fact, the *parS* sequence is only 1-kb-long and could potentially be shortened to 200 bp (NeoVirtech, personal communication). In addition, several reports in yeast and animal cells have already demonstrated the innocuity of the ANCHOR system to endogenous processes such as transcription and replication (Germier et al., 2018). This particularity makes the ANCHOR system very suitable to monitor single-copy genes in its native genomic environment. In this study, ANCHOR lines were generated by T-DNA insertion. Five out of eight independent lines showed strong ANCHOR signals. This could indicate that ANCHOR insertion site is important to be functional. However, we cannot conclude whether the ANCHOR system is suitable to monitor a genomic locus located in a heterochromatic environment. The absence of *parS*-ParB:GFP *foci* could in fact be a consequence of a lack of ParB:GFP expression, which does not mean that *parS* accessibility is compromised. Having a separate transgene for ParB:GFP expression and *parS* detection would be necessary to address this point. In addition, T-DNA transgenes and *Agrobacterium*-directed transformation can be a source of genomic and epigenomic instability, both in *cis* and in *trans* (Rajeevkumar et al., 2015). Moreover, they can also modify the nuclear architecture of their insertion site (Grob and Grossniklaus, 2019). To specifically monitor the dynamics of selected single loci, the *parS* sequence would need to be inserted at a precise position within the desired locus. A recent approach that combine CRISPR-Cas9 technology and a homologous recombination-donor cassette can generate knock-in *A. thalian*a plants (Miki et al., 2018; Wolter et al., 2018; Merker et al., 2020). The implementation of the *parS* knock-in strategy will really improve the innocuity of this approach on the local chromatin state and should strongly reduce any bias on its nuclear positioning.

Another advantage of the ANCHOR approach is the possibility to use simultaneously different combinations of *parS*-ParB. In fact, ParB binding on *parS* sequence is species-specific, and several combinations have successfully been used separately or simultaneously so far. In this study, we used a specific *parS*-ParB, but an additional specific combination could be used. In theory, up to three combinations could be used simultaneously (Saad et al., 2014, NeoVirTech peronnal communication), although an important preliminary work would be required for plant material preparation. For instance, two alleles from the same gene could be differently labeled to monitor their potential associations while being expressed or silenced. This is an important question since previous observations suggest that allele aggregation could participate in gene transcriptional regulation (Rosa et al., 2013). These color combinations could also be used to follow the distance of two proximal regions during DNA repair, for example, as already shown in yeast (Saad et al., 2014) or to label borders of a genomic regions that can undergo different chromatin states during stress or development. This system will provide a useful tool to study the spatial organization and the dynamic behavior of chromatin at the single locus level.

## Data Availability Statement

The sequencing data presented in this study are not readily available due to proprietary restrictions. The remaining original contributions presented in the study are included in the article/Supplementary Material, and further inquiries can be directed to the corresponding author.

## Author Contributions

MI, FP, and SR designed the experiments. AM, MI, CP, and FP performed the experiments. AM, MI, NP, SR, and FP analyzed the data. SD, FG, KB, and MM participated in material preparation or analyzing tools. FP wrote the paper and acquired main funding. SR edited the paper. All authors contributed to the article and approved the submitted version.

## Conflict of Interest

FG is an employee and FG and KB are shareholder of NeoVirTech. NeoVirTech did not have any scientific or financial contribution to this study. ANCHOR system is the property of NeoVirTech SAS, Toulouse, France. The remaining authors declare that the research was conducted in the absence of any commercial or financial relationships that could be construed as a potential conflict of interest.

## Supplementary Material

Supplementary Figure 1 Chromatin states flanking the insertion site in T2F ANCHOR line. **(A)** Snapshot of the chromatin states enriched in the region flanking the transgene insertion site in the line T2F (https://jbrowse.arabidopsis.org/). (**B)** Histogram representing the relative enrichment of each chromatin state in the 5 kb upstream and downstream region of the transgene insertion site in the line T2F.

Supplementary Figure 2 Detection of *parS*-ParB foci in cells with different ploidy levels. Detection of *parS*-ParB:GFP foci (green) in fixed and sorted nuclei according to their ploidy levels by fluorescent-assisted cell sorting. Nuclear DNA is labeled with DAPI (gray).

Supplementary Figure 3 Pollen grain and trichome cell. Confocal images of the *parS-*ParB:GFP signal in a trichome cell (top panels) or in pollen grains (bottom panels). Images on the right are saturated to show the trichome contour or the pollen grains.

Supplementary Figure 4 Co-localization of *parS*-ParB foci with H3Ac and H3K27me3 marks. Detection of *parS*-ParB:GFP foci (green) and posttranslationally modified histones (red) in fixed and isolated nuclei *from A. thaliana* Col-0 plants T2F. Nuclear DNA is labeled with DAPI (gray). Trimethylated H3K27 signals are shown in the (**A)**, while acetylated H3 are shown in (**B). (C,D)** show the relative intensity of each signal.

## References

[B1] Arai R. Sugawara T. Sato Y. Minakuchi Y. Toyoda A. Nabeshima K.. (2017). Reduction in chromosome mobility accompanies nuclear organization during early embryogenesis in *Caenorhabditis elegans*. Sci. Rep. 7:3631. 10.1038/s41598-017-03483-5 28623274 PMC5473868

[B2] Blanco-Rodriguez G. Gazi A. Monel B. Frabetti S. Scoca V. Mueller F.. (2020). Remodeling of the core leads HIV-1 preintegration complex into the nucleus of human lymphocytes. J. Virol. 94. 10.1128/JVI.00135-20 PMC7269431 32238582

[B3] Clough S. J. Bent A. F. (1998). Floral dip: a simplified method for Agrobacterium-mediated transformation of *Arabidopsis thaliana*. Plant J. 16, 735–743. 10.1046/j.1365-313x.1998.00343.x 10069079

[B4] Dubarry N. Pasta F. Lane D. (2006). ParABS systems of the four replicons of *Burkholderia cenocepacia*: new chromosome centromeres confer partition specificity. J. Bacteriol. 188, 1489–1496. 10.1128/JB.188.4.1489-1496.2006 16452432 PMC1367244

[B5] Dumur T. Duncan S. Graumann K. Desset S. Randall R. S. Scheid O. M.. (2019). Probing the 3D architecture of the plant nucleus with microscopy approaches: challenges and solutions. Nucleus 10, 181–212. 10.1080/19491034.2019.1644592 31362571 PMC6682351

[B6] Durut N. Abou-Ellail M. Pontvianne F. Das S. Kojima H. Ukai S.. (2014). A duplicated NUCLEOLIN gene with antagonistic activity is required for chromatin organization of silent 45S rDNA in Arabidopsis. Plant Cell 26, 1330–1344. 10.1105/tpc.114.123893 24668745 PMC4001387

[B7] Earley K. W. Haag J. R. Pontes O. Opper K. Juehne T. Song K.. (2006). Gateway-compatible vectors for plant functional genomics and proteomics. Plant J. 45, 616–629. 10.1111/j.1365-313X.2005.02617.x 16441352

[B8] Fang Y. Spector D. L. (2007). Identification of nuclear dicing bodies containing proteins for microRNA biogenesis in living Arabidopsis plants. Curr. Biol. 17, 818–823. 10.1016/j.cub.2007.04.005 17442570 PMC1950788

[B9] Feng C.-M. Qiu Y. Van Buskirk E. K. Yang E. J. Chen M. (2014). Light-regulated gene repositioning in Arabidopsis. Nat. Commun. 5:3027. 10.1038/ncomms4027 PMC4136543 24390011

[B10] Fujimoto S. Sugano S. S. Kuwata K. Osakabe K. Matsunaga S. (2016). Visualization of specific repetitive genomic sequences with fluorescent TALEs in *Arabidopsis thaliana*. J. Exp. Bot. 67, 6101–6110. 10.1093/jxb/erw371 27811079 PMC5100022

[B11] Funnell B. E. (2016). ParB partition proteins: complex formation and spreading at bacterial and plasmid centromeres. Front. Mol. Biosci. 3:44. 10.3389/fmolb.2016.00044 27622187 PMC5002424

[B12] Gallardo F. Schmitt D. Brandely R. Brua C. Silvestre N. Findeli A.. (2020). Fluorescent tagged vaccinia virus genome allows rapid and efficient measurement of oncolytic potential and discovery of oncolytic modulators. Biomedicines 8:543. 10.3390/biomedicines8120543 33256205 PMC7760631

[B13] Germier T. Audibert S. Kocanova S. Lane D. Bystricky K. (2018). Real-time imaging of specific genomic loci in eukaryotic cells using the ANCHOR DNA labelling system. Methods 142, 16–23. 10.1016/j.ymeth.2018.04.008 29660486

[B14] Germier T. Kocanova S. Walther N. Bancaud A. Shaban H. A. Sellou H.. (2017). Real-time imaging of a single gene reveals transcription-initiated local confinement. Biophys. J. 113, 1383–1394. 10.1016/j.bpj.2017.08.014 28978433 PMC5627313

[B15] Grob S. Grossniklaus U. (2019). Invasive DNA elements modify the nuclear architecture of their insertion site by KNOT-linked silencing in *Arabidopsis thaliana*. Genome Biol. 20:120. 10.1186/s13059-019-1722-3 31186073 PMC6560877

[B16] Hinsberger A. Graillot B. Blachère Lopez C. Juliant S. Cerutti M. King L. A.. (2020). Tracing baculovirus AcMNPV infection using a real-time method based on ANCHOR(TM) DNA labeling technology. Viruses 12. 10.3390/v12010050 31906433 PMC7019957

[B17] Kato N. Lam E. (2003). Chromatin of endoreduplicated pavement cells has greater range of movement than that of diploid guard cells in *Arabidopsis thaliana*. J. Cell Sci. 116, 2195–2201. 10.1242/jcs.00437 12692151

[B18] Kearse M. Moir R. Wilson A. Stones-Havas S. Cheung M. Sturrock S.. (2012). Geneious Basic: an integrated and extendable desktop software platform for the organization and analysis of sequence data. Bioinformatics 28, 1647–1649. 10.1093/bioinformatics/bts199 22543367 PMC3371832

[B19] Khosravi S. Dreissig S. Schindele P. Wolter F. Rutten T. Puchta H.. (2020). Live-Cell CRISPR imaging in plant cells with a telomere-specific guide RNA. Methods Mol. Biol. 2166, 343–356. 10.1007/978-1-0716-0712-1_20 32710419

[B20] Komatsu T. Quentin-Froignant C. Carlon-Andres I. Lagadec F. Rayne F. Ragues J.. (2018). *In vivo* labelling of adenovirus DNA identifies chromatin ANCHORing and biphasic genome replication. J. Virol. 92:e00795-18. 10.1128/JVI.00795-18 29997215 PMC6146703

[B21] Lindhout B. I. Fransz P. Tessadori F. Meckel T. Hooykaas P. J. J. van der Zaal B. J. (2007). Live cell imaging of repetitive DNA sequences via GFP-tagged polydactyl zinc finger proteins. Nucleic Acids Res. 35:e107. 10.1093/nar/gkm618 17704126 PMC2018617

[B22] Mariamé B. Kappler-Gratias S. Kappler M. Balor S. Gallardo F. Bystricky K. (2018). Real-time visualization and quantification of human cytomegalovirus replication in living cells using the ANCHOR DNA labeling technology. J. Virol. 92:e00571-18. 10.1128/JVI.00571-18 29950406 PMC6146708

[B23] Matzke A. J. M. Huettel B. van der Winden J. Matzke M. (2005). Use of two-color fluorescence-tagged transgenes to study interphase chromosomes in living plants. Plant Physiol. 139, 1586–1596. 10.1104/pp.105.071068 16339805 PMC1310544

[B24] Matzke M. A. Kanno T. Matzke A. J. M. (2015). RNA-directed DNA methylation: the evolution of a complex epigenetic pathway in flowering plants. Annu. Rev. Plant Biol. 66, 243–267. 10.1146/annurev-arplant-043014-114633 25494460

[B25] Merker L. Schindele P. Puchta H. (2020). Using CRISPR/ttLbCas12a for in planta gene targeting in *A. thaliana*. Curr. Protoc. Plant Biol. 5:e20117. 10.1002/cppb.20117 32865887

[B26] Meschichi A. Rosa S. (2021). Visualizing and measuring single locus dynamics in *Arabidopsis thaliana*. Methods Mol. Biol. 2200, 213–224. 10.1007/978-1-0716-0880-7_10 33175380

[B27] Meshorer E. Yellajoshula D. George E. Scambler P. J. Brown D. T. Misteli T. (2006). Hyperdynamic plasticity of chromatin proteins in pluripotent embryonic stem cells. Dev. Cell 10, 105–116. 10.1016/j.devcel.2005.10.017 16399082 PMC1868458

[B28] Miki D. Zhang W. Zeng W. Feng Z. Zhu J.-K. (2018). CRISPR/Cas9-mediated gene targeting in Arabidopsis using sequential transformation. Nat. Commun. 9:1967. 10.1038/s41467-018-04416-0 29773790 PMC5958078

[B29] Nguyen H. Q. Bosco G. (2015). Gene positioning effects on expression in eukaryotes. Annu. Rev. Genet. 49, 627–646. 10.1146/annurev-genet-112414-055008 26436457

[B30] Picart-Picolo A. Grob S. Picault N. Franek M. Llauro C. Halter T.. (2020). Large tandem duplications affect gene expression, 3D organization, and plant-pathogen response. Genome Res. 30, 1583–1592. 10.1101/gr.261586.120 33033057 PMC7605254

[B31] Pontvianne F. Blevins T. Chandrasekhara C. Feng W. Stroud H. Jacobsen S. E.. (2012). Histone methyltransferases regulating rRNA gene dose and dosage control in Arabidopsis. Genes Dev. 26, 945–957. 10.1101/gad.182865.111 22549957 PMC3347792

[B32] Pontvianne F. Boyer-Clavel M. Saez-Vasquez J. (2016). Fluorescence-activated nucleolus sorting in Arabidopsis. Methods Mol. Biol. 1455, 203–211. 10.1007/978-1-4939-3792-9_15 27576720

[B33] Pontvianne F. Grob S. (2020). Three-dimensional nuclear organization in *Arabidopsis thaliana*. J. Plant Res. 133, 479–488. 10.1007/s10265-020-01185-0 32240449

[B34] Pontvianne F. Liu C. (2019). Chromatin domains in space and their functional implications. Curr. Opin. Plant Biol. 54, 1–10. 10.1016/j.pbi.2019.11.005 31881292

[B35] Rajeevkumar S. Anunanthini P. Sathishkumar R. (2015). Epigenetic silencing in transgenic plants. Front. Plant Sci. 6:693. 10.3389/fpls.2015.00693 26442010 PMC4564723

[B36] Rosa S. De Lucia F. Mylne J. S. Zhu D. Ohmido N. Pendle A.. (2013). Physical clustering of FLC alleles during Polycomb-mediated epigenetic silencing in vernalization. Genes Dev. 27, 1845–1850. 10.1101/gad.221713.113 24013499 PMC3778238

[B37] Saad H. Gallardo F. Dalvai M. Tanguy-le-Gac N. Lane D. Bystricky K. (2014). DNA dynamics during early double-strand break processing revealed by non-intrusive imaging of living cells. PLoS Genet. 10:e1004187. 10.1371/journal.pgen.1004187 24625580 PMC3952824

[B38] Santos A. P. Gaudin V. Mozgová I. Pontvianne F. Schubert D. Tek A. L.. (2020). Tiding-up the plant nuclear space: domains, function and dynamics. J. Exp. Bot. 71, 5160–5178. 10.1093/jxb/eraa282 32556244 PMC8604271

[B39] Seeber A. Hauer M. H. Gasser S. M. (2018). Chromosome dynamics in response to DNA damage. Annu. Rev. Genet. 52, 295–319. 10.1146/annurev-genet-120417-031334 30208290

[B40] Sequeira-Mendes J. Araguez I. Peiro R. Mendez-Giraldez R. Zhang X. Jacobsen S. E.. (2014). The functional topography of the arabidopsis genome is organized in a reduced number of linear motifs of chromatin states. Plant Cell 26, 2351–2366. 10.1105/tpc.114.124578 24934173 PMC4114938

[B41] Shaban H. A. Seeber A. (2020). Monitoring global chromatin dynamics in response to DNA damage. Mutat. Res. 821:111707. 10.1016/j.mrfmmm.2020.111707 32505939

[B42] Watanabe K. Pecinka A. Meister A. Schubert I. Lam E. (2005). DNA hypomethylation reduces homologous pairing of inserted tandem repeat arrays in somatic nuclei of *Arabidopsis thaliana*. Plant J. 44, 531–540. 10.1111/j.1365-313X.2005.02546.x 16262704

[B43] Wolter F. Klemm J. Puchta H. (2018). Efficient in planta gene targeting in Arabidopsis using egg cell-specific expression of the Cas9 nuclease of *Staphylococcus aureus*. Plant J. 94, 735–746. 10.1111/tpj.13893 29573495

[B44] Yelagandula R. Stroud H. Holec S. Zhou K. Feng S. Zhong X.. (2014). The histone variant H2A.W defines heterochromatin and promotes chromatin condensation in Arabidopsis. Cell 158, 98–109. 10.1016/j.cell.2014.06.006 24995981 PMC4671829

