# Supplementary figures and images for: ANCHOR: A Technical Approach to Monitor Single-Copy Locus Localization in Planta
Source: Front Plant Sci. 2021 Jul 6;12:677849. doi: 10.3389/fpls.2021.677849 (PMC8290188; doi:10.3389/fpls.2021.677849)

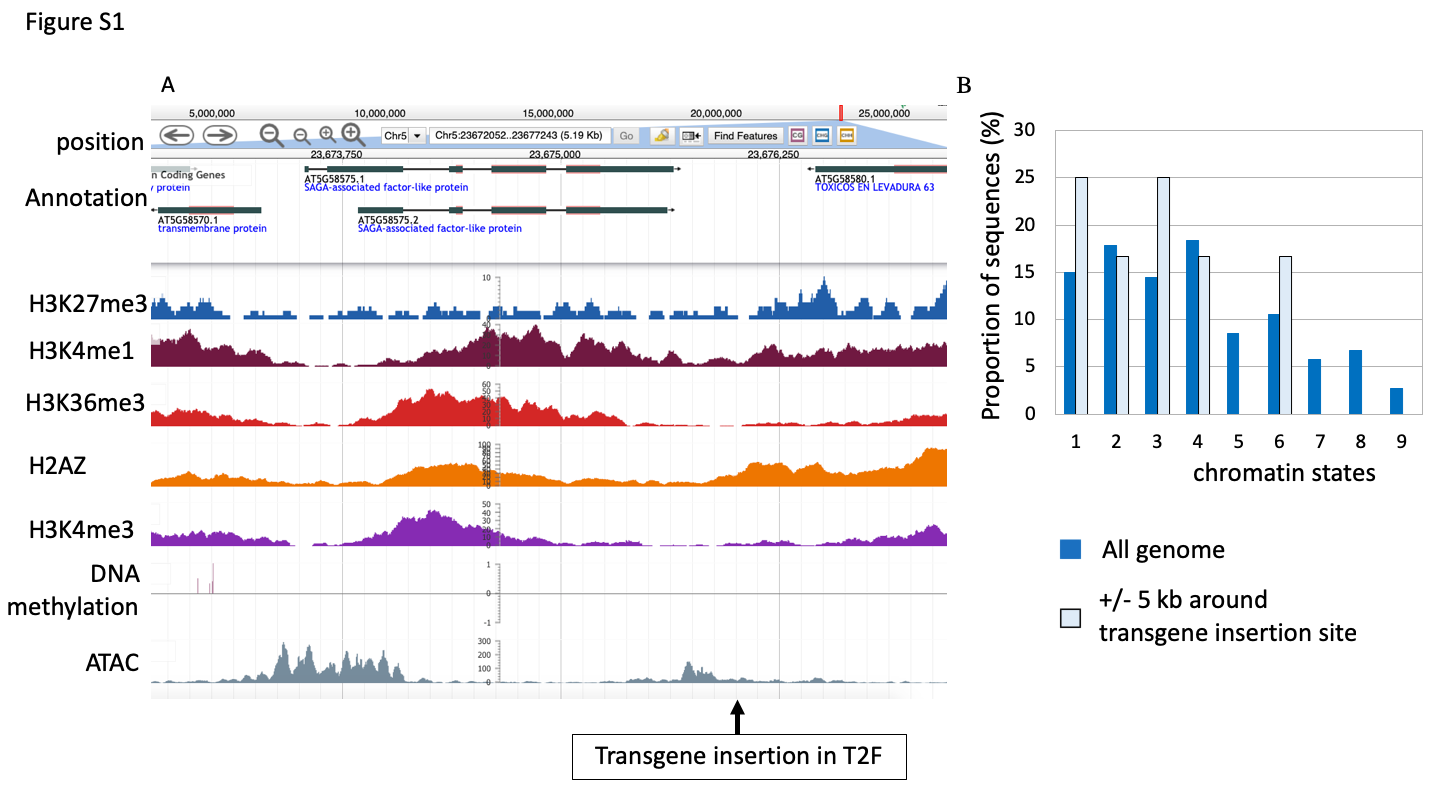

Supplement: Supplementary Figure 1 — Chromatin states flanking the insertion site in T2F ANCHOR line. (A) Snapshot of the chromatin states enriched in the region flanking the transgene insertion site in the line T2F (https://jbrowse.arabidopsis.org/). (B) Histogram representing the relative enrichment of each chromatin state in the 5 kb upstream and downstream region of the transgene insertion site in the line T2F. [file Image_1.TIFF]

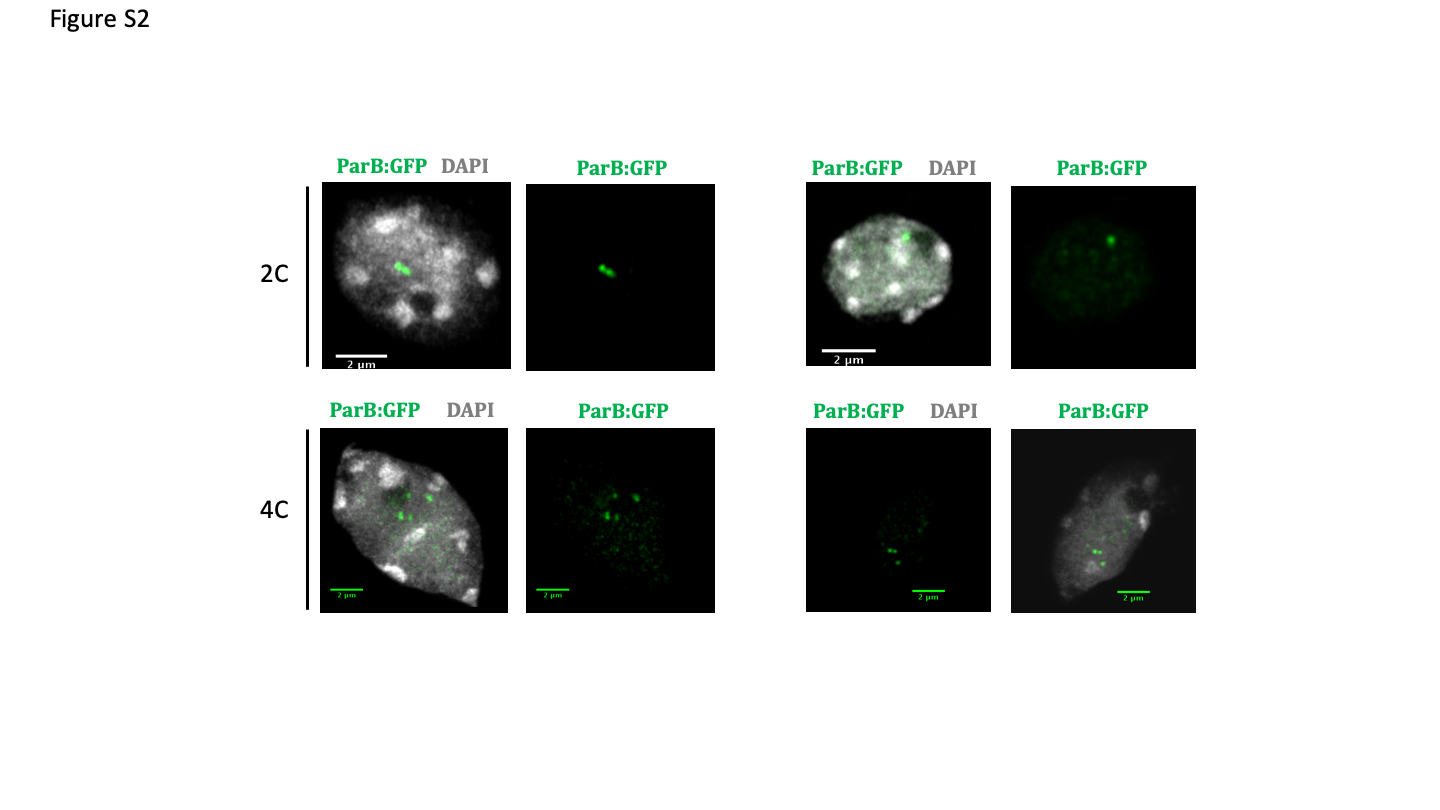

Supplement: Supplementary Figure 2 — Detection of parS-ParB foci in cells with different ploidy levels. Detection of parS-ParB:GFP foci (green) in fixed and sorted nuclei according to their ploidy levels by fluorescent-assisted cell sorting. Nuclear DNA is labeled with DAPI (gray). [file Image_2.TIFF]

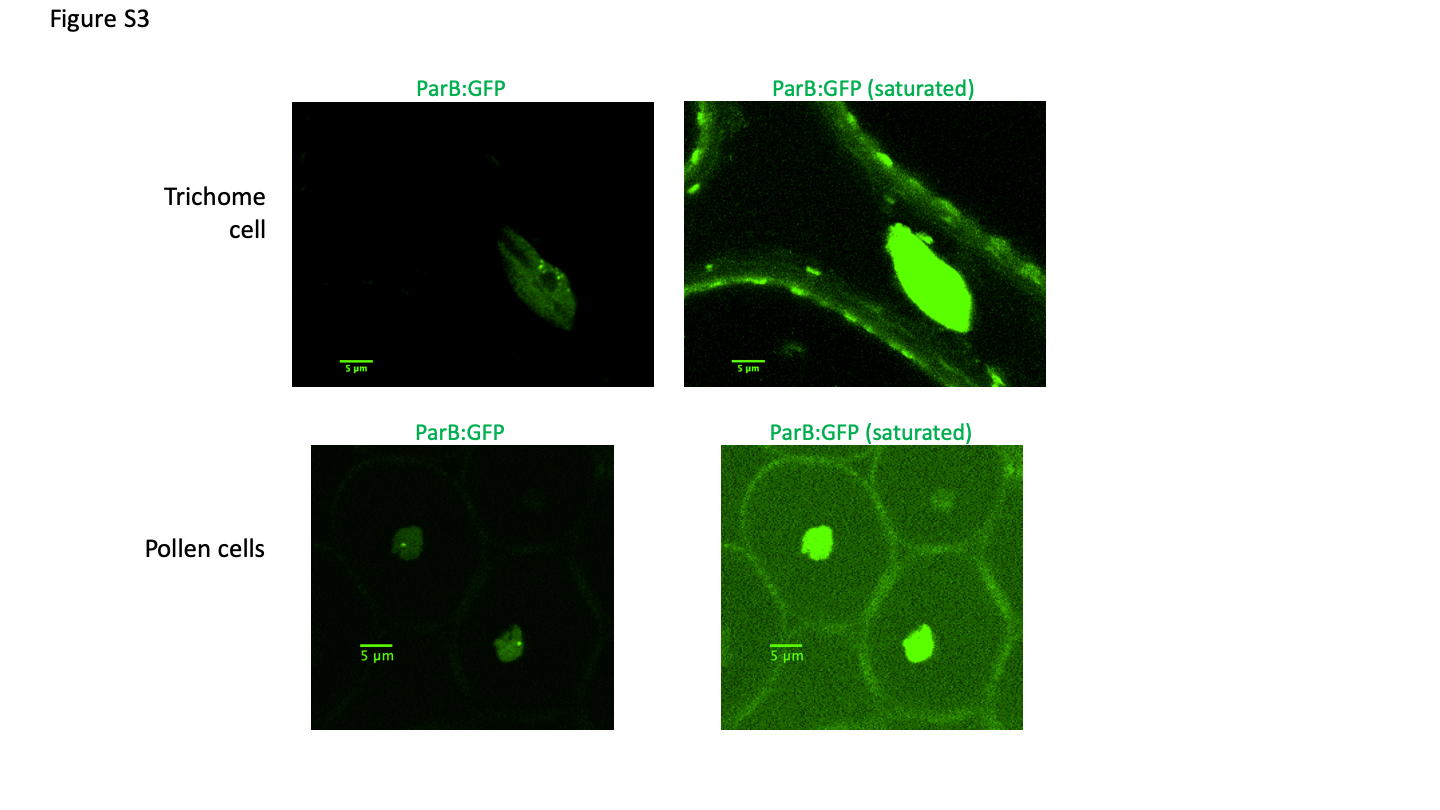

Supplement: Supplementary Figure 3 — Pollen grain and trichome cell. Confocal images of the parS-ParB:GFP signal in a trichome cell (top panels) or in pollen grains (bottom panels). Images on the right are saturated to show the trichome contour or the pollen grains. [file Image_3.TIFF]

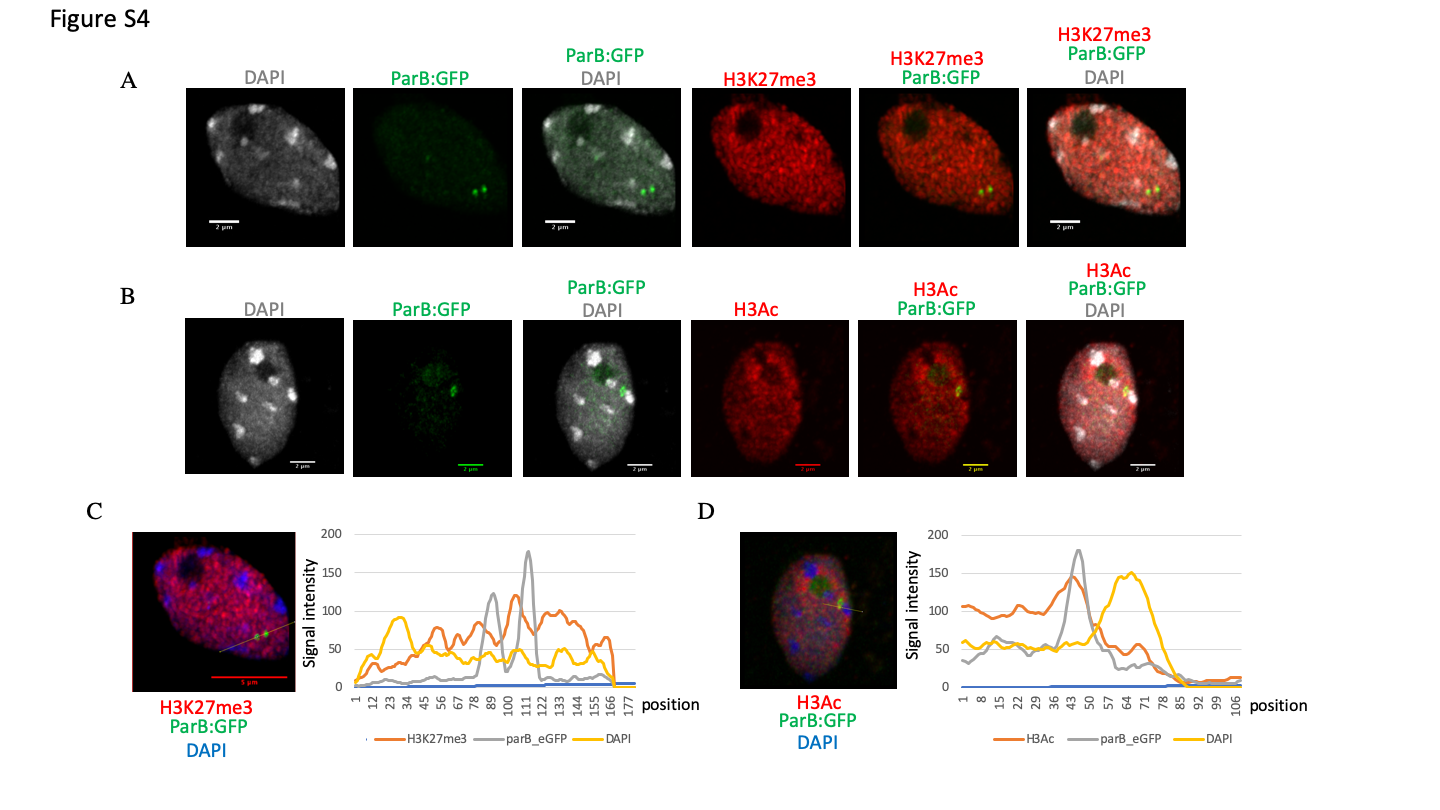

Supplement: Supplementary Figure 4 — Co-localization of parS-ParB foci with H3Ac and H3K27me3 marks. Detection of parS-ParB:GFP foci (green) and posttranslationally modified histones (red) in fixed and isolated nuclei from A. thaliana Col-0 plants T2F. Nuclear DNA is labeled with DAPI (gray). Trimethylated H3K27 signals are shown in the (A), while acetylated H3 are shown in (B). (C,D) show the relative intensity of each signal. [file Image_4.TIFF]
